# Homuncular mirrors: misunderstanding causality in embodied cognition

**DOI:** 10.3389/fnhum.2014.00299

**Published:** 2014-05-13

**Authors:** Ezequiel P. Mikulan, Lucila Reynaldo, Agustín Ibáñez

**Affiliations:** ^1^Laboratory of Experimental Psychology and Neuroscience, Institute of Cognitive Neurology, Favaloro UniversityBuenos Aires, Argentina; ^2^UDP-INECO Foundation Core on Neuroscience, Diego Portales UniversitySantiago, Chile; ^3^National Scientific and Technical Research CouncilBuenos Aires, Argentina; ^4^Department of Psychology, Universidad Autónoma del CaribeBarranquilla, Colombia

**Keywords:** embodied cognition, mirror neuron system, networks, language understanding, causality

Emerging theories on embodied cognition have caused high expectations, ambitious promises, and strong controversies. Several criticisms have been explained elsewhere (Mahon and Caramazza, 2008; Cardona et al., 2014) and will not be discussed further here. In this paper, we will focus on a specific explanatory strategy frequently assessed by the radical embodied cognition approaches: the use of homuncular explanations for the explicit (or implicit) attribution of causal roles in the comprehension of language understanding. We first present this criticism regarding a prototypical example: the mirror neuron system (MNS) (Rizzolatti and Craighero, 2004; Iacoboni and Dapretto, 2006) in the field of language understanding and then extend our conclusions to other programs of embodied cognition. Here we discuss the radical claims that propose the MNS as the putative mechanism for multiple cognitive and social psychology constructs (e.g., Gallese, 2008; Cattaneo and Rizzolatti, 2009; Iacoboni, 2009) and the critical role of the MNS in language understanding (Heyes, 2010a; Hickok, 2013).

## A big problem: homuncularity and causality of the MNS

In the homuncular explanation (Clark, 1997; Kolak et al., 2006), a phenomenological description of a cognitive event attributed to a whole person (in this case, language understanding) is granted to a subset of brain regions (in this case, the MNS) by using discrete representations. This is the case for radical MNS accounts. The MNS helps in understanding observed actions by extracting and representing goals or meanings (Rizzolatti et al., 2001; Rizzolatti and Craighero, 2004). The fundamental role proposed for the MNS is that of allowing the individual to understand the goal of the action he/she is observing (Fogassi et al., 2005). Gallese (2006) proposed that the MNS allows one to directly access the understanding of others. The so-called “direct-matching hypothesis” suggests that “an action is understood when its observation causes the motor system of the observer to ‘resonate’” (Rizzolatti et al., 2001). Thus, the MNS is proposed as an automatic and mandatory mechanism for understanding (Csibra, 2007).

These “homuncular” approaches to the MNS have favored a plethora of mesmerizing functional explanations, from action to higher social cognition (Heyes, 2010b). In the case of language, the intrinsically linguistic property of “understanding” becomes a property of MNS activation. Contrary to homuncular explanations, current brain network approaches to language (Turken and Dronkers, 2011; Friederici and Gierhan, 2013) have shown that language processing requires an orchestrated coordination of different brain regions indexing different processes. The MNS probably plays an important role in priming or facilitating understanding (or even perhaps in indexing action semantics), but this does not imply that the MNS plays a key role in language *understanding*. Even in action language processing, where the MNS seems to be more engaged, other non-MNS regions (such as specific sites for language processing and motor habits) seem to play an important role (Arbib, 2010; van Dam et al., 2010; Amoruso et al., 2013; Cardona et al., 2013; Ibanez et al., 2013; Sakreida et al., 2013). Thus, a single MNS process explaining the whole phenomenon of understanding seems to be a less fruitful approach when compared with a network view of language processing.

The homuncular explanation attributes a causal role to a specific region regarding a complete function. In this radical approach, instead of considering the MNS as an important hub of a network indexing language properties, the MNS itself seems to generate language understanding. Several radical claims highlighting this causal mechanism in language understanding have been proposed. For example, Pulvermüller (2005a) wrote: “… words that denote internal states, such as ‘pain’ or ‘disgust,’ can be understood *only* because both speaker and listener can relate them to similar motor programs…” (italics mine); and furthermore: “understanding language means relating language to one's own actions.” Aziz-Zadeh et al. (2006) declare: “these results suggest a key role of mirror neuron areas in the re-enactment of sensory-motor representations during conceptual processing of actions invoked by linguistic stimuli” (see also Zarr et al., 2013).

Considering the MNS as a causal explanatory mechanism for language understanding would appear like a pseudo-explanation. The homuncular, metonymic attribution of language understanding as a causal property of the MNS involves nothing but a lack of explanation. In spite of these radical claims about the MNS, to our knowledge there is no canonical or putative mechanistic explanation for language understanding based on the MNS. By definition, the MNS contains mirror neurons and other neurons for matching the observation and execution of action (Rizzolatti and Craighero, 2004; Iacoboni and Dapretto, 2006). How does the MNS generate or produce understanding? Just by resonating when observing or executing actions? The MNS property of being activated when observing/executing actions is not an explanation of how language understanding emerges. At the very least, language understanding requires syntactic and semantic access, memory, executive functions, and other language-specific knowledge (Binder et al., 1997; Friederici, 2011; Price, 2012). A subset of neurons in an artificial system can easily be trained to respond to action observation/execution, mimicking the basic definition of the MNS. Nevertheless, this property by itself will surely not generate language understanding. The main problem with the explanation of language understanding as MNS activity is that there is no real explanation at the level of language content.

Is MNS activation a cause or an accompanying effect of language understanding? There is a lack of empirical evidence for the putative causal role of MNS in language understanding. In cognitive neuroscience, there are illustrative examples of the causal role of an area in a function. For example, electrical stimulation of the anterior insula triggers the experience of disgust (Caruana et al., 2011). Similarly, electrical stimulation of the fusiform gyrus can selectively disrupt face perception (Parvizi et al., 2012). Therefore, we can conclude that the insula and the fusiform gyrus have a causal and critical role in the experience of disgust and in face perception, respectively. Those cases do not have a full causal explanation (in the Aristotelian sense of an “efficient” cause) because these regions are connected with several other brain regions whose involvement also affects the emotional and perceptual response. Nevertheless, it is still possible to suggest a critical role of these regions in the generation of the disgust experience or in facial perception.

Focal lesion studies may provide more direct answers to these questions (Rorden and Karnath, 2004). Reports on aphasic and apraxic patients fully support the embodied nature of cognition. However, these have yielded controversial results regarding the causal explanations of “understanding.” Overlaps (Rothi et al., 1985; Saygin et al., 2003; Nelissen et al., 2010) and dissociations (Rothi et al., 1991; Mahon and Caramazza, 2005) between language and action networks have been reported. In any case, the overlap is not enough to assert that understanding occurs as an effect of motor resonance or to establish a unidirectional causal explanation. Experiments in which researchers are able to show a given region's critical role in a specific function are extremely scarce in MNS research regarding language understanding. To our knowledge, there is no single experiment demonstrating that MNS activity plays a causal role in language understanding instead of merely reflecting it. Thus, the strong claims about the causal role of the MNS in language understanding contrast with the scarce available evidence.

Most of the evidence regarding the MNS and action language is centered around facilitation effects, i.e., understanding is not dependent on MNS activation (measured directly or indirectly), but is only facilitated by it. For example, Pulvermüller et al. (2005b) showed that Transcranial Magnetic Stimulation (TMS) of the hand area in the left hemisphere led to faster responses to hand-related words in a lexical decision task, while stimulating the leg area in the left hemisphere had the same effect on leg-related words. This effect was not present in control conditions (stimulating the right hemisphere and sham stimulation). Similarly, Tucker and Ellis (2004) found a response compatibility effect when subjects used an input device that required either a power or a precision grip to indicate whether objects that required either type of grip were natural or man-made. Responses were faster when the presented object (picture or word) required the same grip type as the input device. Most studies show language understanding as capacity that is facilitated by MNS involvement or attenuated by MNS disruption. Nevertheless, no studies have assessed interfered or abolished understanding, or shown a critical dependence on the MNS. Thus, evidence suggests that the MNS reflects the effect of understanding rather than causing it (Hickok, 2013). The MNS might play an important role in general associative learning (Heyes, 2010b; Cooper et al., 2013) or a specific facilitation/priming effect in language understanding, but not a causal role in understanding by itself. There is no doubt about the activation of the MNS during execution and observation, but several concerns arise when this activation is interpreted as a causal explanatory mechanism in several cognitive domains.

## Causal explanations in neuroscientific embodied cognition

The notion of a causal role for MNS in language understanding is a prototypical example of a radical claim that a single region subserves understanding. In the language domain, other similar causal explanations have been proposed. The Embodied Semantics theory claims that processing the meaning of a concept recruits the same neural networks that underlie the perceptual and motor experiences associated with it (Gallese and Lakoff, 2005). In other words, regions that are activated during action observation and action execution should also be activated during the comprehension of words referring to those actions. It has been reported that this activation follows a somatotopical pattern (Hauk et al., 2004; Pulvermüller, 2005a), that is, leg concepts (“kicking”) activate the homuncular leg area in the motor cortex and mouth concepts (“eating”) activate the mouth area. Even though evidence has shown this type of activation pattern, the match is not exact and the overlap is inconsistent within and across different studies (Postle et al., 2008; Turella et al., 2009; Arbib, 2010; Fernandino and Iacoboni, 2010; Arevalo et al., 2012). Other regions that have been implicated in tasks involving the processing of linguistic stimuli are the prefrontal cortex, the temporal lobe and the cerebellum (Arbib, 2010). Furthermore, lesions to the motor cortex do not necessarily cause deficits in action-word processing (Saygin et al., 2004). In sum, although there is motor and premotor activation when processing language (Glenberg et al., 2008) and linguistic comprehension might be enhanced by it, there is no conclusive empirical evidence showing that this is a sufficient mechanism for linguistic understanding (Fischer and Zwaan, 2008).

Other areas of embodied cognition, including radical MNS approaches to action understanding, imitation, emotion, and social cognition, present the same potential pitfall: the temptation to use a simplistic homuncular explanation for the phenomenon of understanding through a single resonating brain area. Current brain network views and non-MNS accounts of classical domains such as action observation/recognition (Buccino et al., 2004; Kokal et al., 2009), imitation (Molenberghs et al., 2009), language (Grodzinsky et al., 2000; Hickok and Poeppel, 2007; Friederici, 2011) and social cognition (emotion, empathy, and theory of mind; Baird et al., 2011; Decety et al., 2012; Ibanez and Manes, 2012; Kennedy and Adolphs, 2012) can be integrated with the experience-based and situated nature of cognition without appealing to a simplistic execution-observation matching system or attributing the cognitive phenomenon of interest to a single brain region. Although our experience is embodied, our emotions are embodied, and even our culture is embodied, this does not mean *ipso facto* that the activation of discrete hypothetical representations in a single region would be enough to explain the emergence of understanding. In other words, emotions, language and culture are grounded (Barsalou, 2008) in our bodily experiences, but this does not necessarily mean that there is a simple isomorphism between the actual body and the spatiotemporally-distributed activity of body signals in the brain (Berlucchi and Aglioti, 2010).

The extremely significant emergence of embodied cognition, highlighting the role of the body, emotions and culture as well as the subjective experience in shaping the human mind, can and should be detached from a simplistic and at the same time radical homuncular view that human cognitive understanding is ruled by a single discrete brain area.

### Conflict of interest statement

The authors declare that the research was conducted in the absence of any commercial or financial relationships that could be construed as a potential conflict of interest.
